# Evaluation of clinical effects and plasma metabolomic profiles after the administration of grapiprant in dogs affected by osteoarthritis: a prospective, off–on–off, clinical study

**DOI:** 10.3389/fvets.2026.1811478

**Published:** 2026-06-15

**Authors:** Claudia Piemontese, Chiara Roberta Girelli, Marzia Stabile, Luca Lacitignola, Agata Fraccascia, Antonio Crovace, Francesco Paolo Fanizzi, Francesco Staffieri

**Affiliations:** 1 Department of Precision and Regenerative Medicine and Ionian Area (DIMEPRE-J), University of Bari Aldo Moro, Bari, Italy; 2 Department of Biological and Environmental Sciences and Technologies, University of Salento, Lecce, Italy

**Keywords:** osteoarthritis, grapiprant, pain, inflammation, metabolomic, dog

## Abstract

**Introduction:**

Osteoarthritis (OA) is a prevalent cause of chronic pain and impaired mobility in dogs. Grapiprant, an EP4 prostaglandin receptor antagonist, represents a novel therapeutic option with a favorable safety profile compared to traditional NSAIDs.

**Aim:**

To evaluate the clinical efficacy of grapiprant in dogs with naturally occurring OA and to investigate systemic metabolomic changes using proton nuclear magnetic resonance (^1^H-NMR) spectroscopy.

**Methods:**

Thirty-six dogs with mild to moderate OA were enrolled in a prospective off–on–off study (30 days pre-treatment, 60 days grapiprant treatment at 2 mg/kg, 30 days post-treatment). Outcomes included owner questionnaires (LOAD, CBPI), pressure-sensitive walkway gait analysis (GLS), orthopedic evaluation, and plasma metabolomics.

**Results:**

Grapiprant significantly reduced LOAD scores at T30 and T60 compared with baseline (*p < 0.05), with partial regression at Tpost. GLS improved significantly at T60 (**p < 0.01). Metabolomic analysis (both unsupervised and supervised methods) highlighted a clear discrimination between pre-and post-treatment plasma samples, revealing a statistically significant decrease in lactate, N-acetyl glycoproteins and formate in pretreatments samples and, on the contrary a significantly increase in citrate (*p* < 0.05).

**Conclusion:**

Grapiprant improved mobility and pain control in dogs with OA and induced metabolomic shifts consistent with reduced inflammation and improved energy metabolism. This is the first study to integrate clinical outcomes with metabolomics in canine OA, supporting the translational value of metabolic biomarkers for monitoring therapeutic response.

## Introduction

Osteoarthritis (OA) is one of the most common chronic musculoskeletal disorders in dogs, affecting up to 20% of the population over one year of age (1, 2). It is a progressive, degenerative disease involving the entire joint organ, including cartilage, subchondral bone, synovium, and periarticular structures. Clinical signs include pain, stiffness, reduced mobility, and impaired quality of life. Large breed dogs are particularly predisposed, and contributing factors include genetic background, trauma, obesity, and aging (1–3). Increasing evidence indicates that OA is not only a degenerative process but also a condition characterized by low-grade chronic inflammation and metabolic dysregulation that perpetuate joint damage and functional decline (4). Pharmacological management of canine OA largely relies on non-steroidal anti-inflammatory drugs (NSAIDs), which provide analgesia and improved mobility but may cause gastrointestinal, renal, or hepatic side effects during long-term use (5). Grapiprant, an antagonist of the prostaglandin E2 (PGE2) EP4 receptor, represents the first drug of a new class known as piprants. The EP4 receptor is a key mediator of PGE2-driven pain and inflammation; thus, its selective blockade offers a targeted mechanism with a favorable safety profile compared to conventional NSAIDs (6, 7). Randomized clinical trials have demonstrated the efficacy of grapiprant in reducing pain and improving mobility in dogs with naturally occurring OA, with sustained tolerability even at supra-therapeutic doses (8, 9).

In parallel, metabolomics has emerged as a powerful tool to investigate the biochemical alterations underlying OA (10). Using proton nuclear magnetic resonance (^1^H-NMR) spectroscopy, metabolomic profiling of biological fluids such as plasma and synovial fluid provides a comprehensive overview of systemic and local metabolic changes (11). In both human and veterinary medicine, OA has been associated with altered concentrations of amino acids, lipids, lactate, and energy-related metabolites, reflecting mitochondrial dysfunction, hypoxia, and inflammatory status (11–13). Synovial fluid, being in direct contact with joint tissues, is considered the most informative matrix; however, its collection is often limited in chronic OA. Plasma, on the other hand, is more easily accessible and may serve as a surrogate to monitor systemic metabolic shifts and treatment responses (12, 14). While clinical scoring systems provide valuable information on pain and functional impairment, they do not capture the underlying biological processes. Integrating metabolomic profiling may therefore offer complementary insights by linking clinical outcomes with systemic metabolic alterations and improving the identification of objective biomarkers of treatment response.

Despite growing interest, very few studies have explored how pharmacological interventions affect the metabolomic profile of OA patients. Pharmacometabolomics offers the opportunity to capture drug-induced metabolic changes, identify biomarkers of therapeutic response, and stratify patients according to metabolic phenotypes (15). In veterinary medicine, this approach remains largely unexplored, and no data are currently available on the impact of grapiprant on systemic or joint metabolism. Beyond its veterinary relevance, this approach may also contribute to translational research by supporting the identification of metabolic biomarkers of treatment response and advancing precision medicine strategies in osteoarthritis.

Although clinical efficacy of grapiprant has been previously described, its effects on systemic metabolic pathways remain largely unexplored. In particular, there is a lack of data linking clinical improvement to underlying metabolic changes in dogs with naturally occurring osteoarthritis. Addressing this gap may provide novel insights into the mechanisms of action of grapiprant and support the identification of objective biomarkers of treatment response.

The present prospective, within-subject, off–on–off clinical study aimed to assess the clinical efficacy of grapiprant in dogs with mild to moderate OA and to investigate its effects on plasma metabolomic profiles. By combining validated owner-reported outcomes, gait analysis, orthopedic assessment, and untargeted ^1^H-NMR metabolomics, this study sought to provide novel insights into the symptomatic and metabolic effects of grapiprant and to evaluate the potential role of metabolomics as a translational tool for monitoring treatment response in canine OA. Activation of the EP4 receptor by prostaglandin E2 is known to promote inflammatory signaling pathways (6, 7), which are in turn associated with metabolic alterations such as increased glycolysis, altered lipid metabolism, and changes in amino acid profiles (16–18). Therefore, selective EP4 receptor antagonism through grapiprant may indirectly modulate systemic metabolic pathways related to inflammation and energy metabolism. On this basis, an untargeted ^1^H-NMR metabolomic approach was used to capture potential treatment-related metabolic changes. We hypothesized that treatment with grapiprant would result in measurable clinical improvement in dogs with osteoarthritis and would be associated with detectable changes in plasma metabolomic profiles.

## Materials and methods

### Study design

This was a prospective within-subject study with an off–on–off design clinical trial conducted at the Veterinary Teaching Hospital, University of Bari, Italy. The study protocol was approved by the Institutional Animal Care and Use Committee of the Department of Emergency and Organ Transplantation (University of Bari, Italy, Prot. N. 032024). Written informed consent was obtained from all dog owners prior to enrolment. The study consisted of three consecutive phases: pre-treatment (30 days, no therapy), treatment (60 days, grapiprant 2 mg/kg PO q24h), and post-treatment (30 days, withdrawal). At the end of each phase, all dogs underwent the same clinical and instrumental evaluations. Clinical, instrumental, and laboratory evaluations were performed at baseline, immediately before starting treatment (Tpre), after 30 (T30) and 60 (T60) days of treatment, and 30 days after treatment discontinuation (Tpost).

### Animals and inclusion criteria

Client-owned dogs (age > 1 yo and weight > 3.6 kg) diagnosed with mild to moderate OA, according to the Canine Osteoarthritis Staging Tool (COAST) (19, 20), were recruited. Inclusion criteria were: owner-reported mobility impairment; clinical evidence of pain in at least one appendicular joint; radiographic signs of OA (articular effusion, osteophytes, subchondral sclerosis, subluxation, erosions/cysts, or intra-articular mineralization); absence of systemic disease; and withdrawal of analgesic/anti-inflammatory treatment for ≥ 4 weeks prior to inclusion. For dogs previously receiving anti-inflammatory medication a washout period of 4 weeks was required. The wash out period was extended on an individual basis if medically required. Dogs should not have received any nutritional supplements, functional foods, or joint diets prior to inclusion. Exclusion criteria were the absence of OA signs, or significant cardiovascular, renal, hepatic, respiratory, neurological, or severe dental disease. A minimum body weight of 3.6 kg was required for inclusion, based on the technical specifications of the pressure-sensitive walkway system (GAIT4 Dog), which may provide less reliable measurements in very small dogs. No dogs were enrolled during an acute flare of the disease; in cases where exacerbations occurred, appropriate treatment was provided to ensure animal welfare, and those conditions were resolved prior to inclusion.

Veterinarian assessment (lameness assessment).

All dogs underwent a complete physical examination at each scheduled visit. All clinical and orthopedic evaluations were performed by the same experienced clinician to ensure consistency across timepoints. The orthopedic examination included the evaluations of posture, mobility, pain at the manipulation of the affected joint and assessment of the range of motion (ROM). The mobility evaluation was performed, at the walk and trot, to classify dogs’ lameness as following: (1) No visible lameness; (2) Temporary: temporary mild/moderate lameness triggered by the physical exercise; (3) Persistent: persistent mild/moderate lameness with episode of acute severe lameness; (4) Severe and Persistent: severe and persistent lameness and/or with the involvement of more than one joint (19).

### Radiographic assessment

The suspected osteoarthritic joints were evaluated with a radiographic examination and where OA diagnosis was confirmed, they were graded based on the following scheme: (1) no signs; (2) Mild: evidence of articular incongruence, possible subchondral sclerosis, absence of osteophytes; (3) Moderate: evidence of articular incongruence, evident subchondral sclerosis, scarce osteophytes; (4) Severe: evidence of articular incongruence, evidence of subchondral sclerosis, presence of osteophytes, bone deformations.

### Owner assessment

At each clinical evaluation the owners were asked to complete the Liverpool Osteoarthritis in Dogs (LOAD) questionnaire (21, 22) and based on the score each patient was assigned to a specific grade of alteration of the mobility: Not affected (0) Mild (< 11) Moderate (11 – 20) Severe (21 – 30), Extreme (31–52). A discomfort grading was also performed asking the owners to give a grade of severity from 1(poor) to 4 (excellent) according to the animal’s status.

The Canine Brief Pain Inventory score (23, 24) was completed by an owner of each dog that was eligible for inclusion in the study. The 4 pain-severity (PS) questions were scored on a scale of 0 (no pain) to 10 (extreme pain). The responses for these questions were averaged to generate the PS score. The 6 pain interference (PI) questions (ie, how much the pain interfered with the dog’s typical function) were scored on a scale of 0 (does not interfere) to 10 (completely interferes). The responses for these questions were averaged to generate the PI score. Quality of life was also scored on a scale from 0 (poor) to 4 (excellent).

*Staging of the OA and inclusion.*

Based on the data collected during the first physical and radiographic examinations the degree of the OA was scored considering the Canine Osteoarthritis Staging Tool (COAST) (19): Pre-Clinical (0–1) At risk (1) Mild (2) Moderate (3) Severe (4). Only dogs classified as stage 2 or 3 were included in the study.

### Computer-assisted GAIT analysis (GAIT4 dog)

A commercially available pressure mat walkway the GAIT4 Dog R walkway (CIR Systems Inc., Sparta, NJ), consisting of a 5.8 × 0.6 m portable mat with 18,432 encapsulated sensors, connected to a dedicated software (GAITFour software version 4.9Wr, CIR Systems Inc., Sparta, NJ, United States), was used to record and evaluate spatial, temporal, and pressure variables for gait analysis. The mat was calibrated by the manufacturer before use. The test was performed at every scheduled visit. Before performing it, each dog was familiarized with the mat for at least 10 min, and then walked on the mat at least 10 times. When possible, the test was performed without leash, to avoid any external influence. The walks recorded were processed by the dedicated software and accurately evaluated by the clinician. Video recordings were reviewed to ensure the right readings. Walks in which the dog exhibited altered behaviors such as stopping, trotting, pulling on leash, turning head significantly, having inconsistent velocity, or less of three gait cycles, were excluded according to the manufacturer advices. The mean of 5 valid and representative walks, consisting in at least three gait cycles, correctly processed, with an internal velocity variability less of 10%, was considered for the analysis for each dog and each time. All tests were supported by a video recording, using a fixed camera placed at 50 centimeters from the floor. Different parameters were selected and evaluated including velocity, GAIT4 Dog Lameness Score (GLS), stance %, Total Pressure Index (TPI), symmetry ratio (forelimb:hindlimb; right:left) (11, 25) The mean velocity was obtained by dividing the distance traveled (in centimeters) by ambulation time (in seconds). GLS was calculated considering weight distribution, based on observed to expected TPI by limb and established body type loading ratios (default 60:40) and should be approximately 100%, considering the absence of lameness values > 95. For the purposes of this study only the GLS score was considered (11).

### Blood sampling and analysis

At baseline and at the end of the treatment phase (T60), plasma was collected. After an overnight fast, 2.5 mL of blood was drawn into EDTA tubes, centrifuged (2000 rpm, 4 °C, 20 min), and 1 mL plasma aliquots were stored at −80 °C.

Plasma was thawed at room temperature, centrifuged (12,000 g, 5 min, 4 °C), and 350 μL of supernatant were mixed with 350 μL phosphate buffer in D₂O (pH 7.4) containing 2 mmol/L NaN₃ and DSS as chemical shift reference. After centrifugation, 600 μL were transferred into 5 mm NMR tubes. The samples were placed in an autosampler (BASC-60 autosampler: Burker Automatic Sample Charger) interfaced with IconNMR version 5 software, and the acquisition of NMR spectra proceeded in automation mode. The ^1^H-NMR spectra were acquired at 300 K, after 5 min for thermal equilibration, on a Bruker Avance III 600 MHz spectrometer (Bruker, Karlsruhe, Germany) with a TCI cryoprobe (inverse triple resonance Cryoprobe Prodigy), a z-axis gradient coil, and automatic tuning-matching (ATM). For each plasma sample, a 1D CPMG (referred to as Carr–Purcell–Meiboom–Gill) spin-echo sequence was applied to suppress signals of large molecules (128 scans, 16 dummy scans, 32 K points, spectral width 20.0276 ppm (12019.230 Hz), relaxation delay 5 s, acquisition time 1.36 s). After acquisition, standard processing procedures were performed using TopSpin 3.6.4 software (Bruker, Biospin, Italy). The resulting Free Induction Decays (FIDs) were multiplied by an exponential weighting function corresponding to a line broadening of 0.3 Hz before Fourier transformation, phase adjustment, and baseline correction. Metabolite assignments were performed using 1D/2D homo- and heteronuclear experiments and confirmed by comparison with literature data (11, 26).

### Data preprocessing and statistical analysis

Data analysis was performed using MedCalc software (Vers. N. 14). The sample size calculation was based on preliminary data obtained prior to the study, considering a 20% change in LOAD and GLS scores between baseline and post-treatment timepoints. The power calculation was performed for a two tailed t test with power of 0.95, an alpha error of 0.05 and an effect size of 2.18, using freely available software (G*Power Version 3.0.10; University of Düsseldorf, Germany) (27). The results of this analysis suggested that a minimum of 15 dogs would be sufficient to detect significant differences between the phases of the study. The sample size calculation indicated a minimum of 15 animals; however, a larger number of dogs was enrolled to increase the robustness of the findings and to account for potential dropouts.

To evaluate changes in clinical parameters (PSS, PIS, QoL, LOAD and GLS) across repeated timepoints, because all the variables were collected from the same subjects at multiple timepoints and Shapiro–Wilk test indicated non-normal distribution, a non-parametric repeated measures strategy was adopted. The Friedman test was applied to assess the overall effect of time. Where a significant main effect was detected, pairwise post-hoc comparisons were performed using the Wilcoxon signed-rank test, with statistical significance set at *p* < 0.05. For descriptive reporting data are presented as median and range (minimum–maximum). For clinical scores (CBPI, LOAD, and GLS), percentage variation from baseline was also calculated to account for inter-individual variability. To evaluate the statistical significance of these percentage variations, Wilcoxon signed-rank test was applied to compare each time point against baseline. Additionally, boxplots were generated to illustrate the distribution of scores across timepoints.

After the spectral data processing, the entire 1H NMR spectra (in the range 10.0–0.9 ppm) were segmented in fixed rectangular buckets of 0.02 ppm width, using the Bruker Amix 3.9.14 (Analysis of Mixture, Bruker BioSpin GmbH, Rheinstetten, Germany) software to transform the NMR spectra into a format suitable for multivariate analysis. As recently reported in the literature (28) the binning spectra into 0.02 ppm segments appears as a quick and very robust choice for the performance of NMR fingerprinting analysis.

The spectral region between 4.90–4.66 ppm was discarded because of the residual peak of water. Moreover, other six spectral regions, were further excluded from the analysis, due to the residual peaks of the anticoagulant ethylenediaminetetraacetic acid (EDTA): (1.20–1.10; 2.58–2.53;2.72–2.68; 3.25–3.06; 4.90–4.66). Centering, normalization to a constant sum, and the Pareto scaling were applied to the bucketed NMR data to improve the performance of statistical analyses (29). Only samples meeting spectral quality criteria and suitable for consistent multivariate analysis were included. The unsupervised principal component analysis (PCA) and the supervised orthogonal partial least squares discriminant analysis (OPLS-DA) was performed to examine the intrinsic variation in the data, using SIMCA 14 software, (Sartorius Stedim Biotech, Umeå, Sweden). The robustness of the statistical models were tested by cross-validation default method (7-fold) and further evaluated with permutation test. The quality of the models (in particular the total variations in the data and the internal cross validation) was described by R^2^ and Q^2^ and p[CV-ANOVA] parameters (16). The model’s predictive ability was confirmed using a misclassification table with Fisher’s exact test. The S-line plot for the pair-wise OPLS-DA model was used to identify the discriminating metabolites (binned NMR signal) responsible for the separation between the sample classes. This tool visualizes the centred loading vector p(ctr) coloured according to the absolute value of the correlation loading, p(corr). The relative change in discriminating metabolites between the classes was evaluated by calculating the mean values +/− standard deviation of selected binned NMR signal (representative of specific NMR unbiased signals for each discriminating metabolite) after spectra normalization (to the total spectrum excluding the residual water region). In particular, the changes in metabolite levels between two groups were calculated as Log2 fold change (FC) ratio of the normalized median intensity of the corresponding signals in the spectra of the groups. The statistical significance of the differences for each variable of the two groups in the pairwise comparison was evaluated using the Student t-test.

## Results

**Figure 1:**
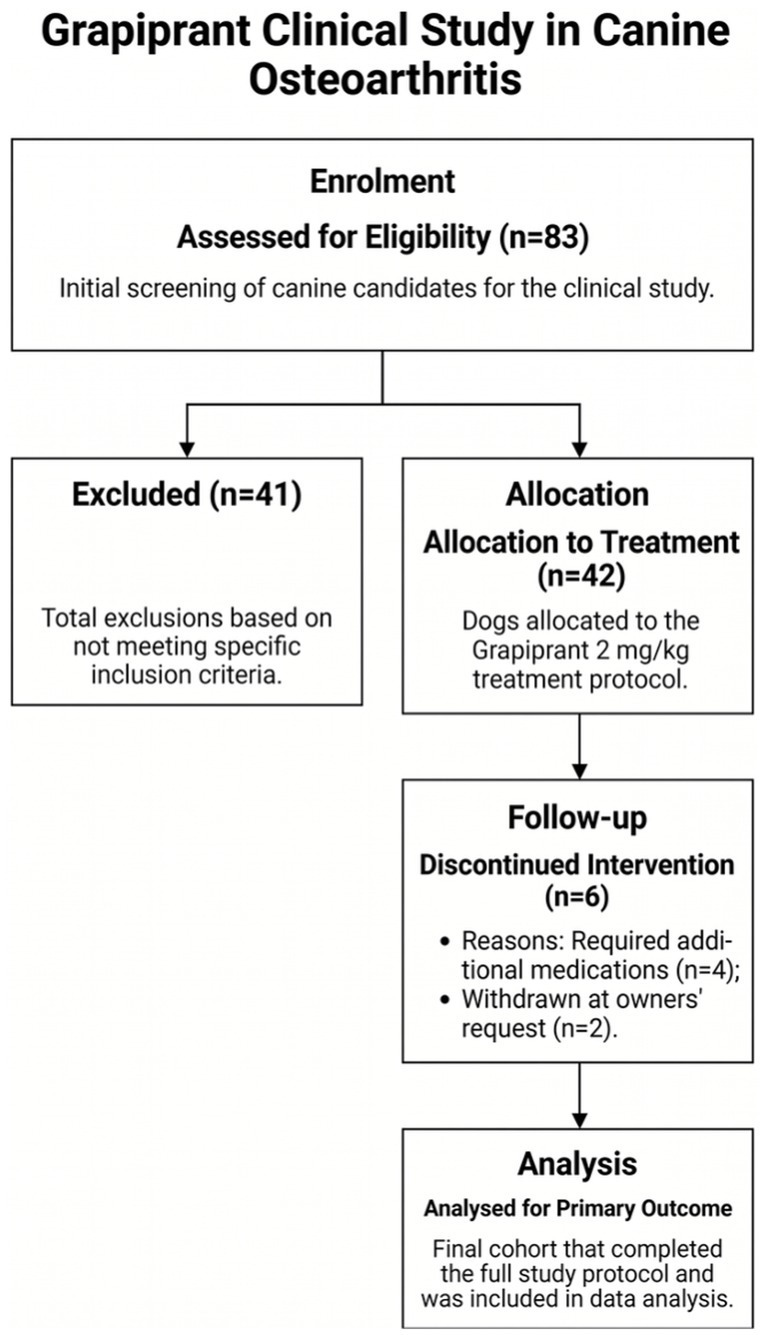
CONSORT flow diagram of the study population, showing the screening, inclusion, exclusion, and follow-up of dogs enrolled in the study across the pre-treatment, treatment, and post-treatment phases.

Eighty-three dogs were initially screened, 42 met all inclusion criteria and were enrolled in the study. During the trial, six dogs were excluded for reasons unrelated to the treatment itself (4 required additional medications and 2 were withdrawn at the owners’ request). A total of 36 dogs completed the study protocol (Supplementary Table S2; Figure 1). No clinically relevant adverse effects were observed during the study period. A flowchart illustrating the screening, inclusion, exclusion, and follow-up of the study population is provided in Figure 1.

**Figure 2:**
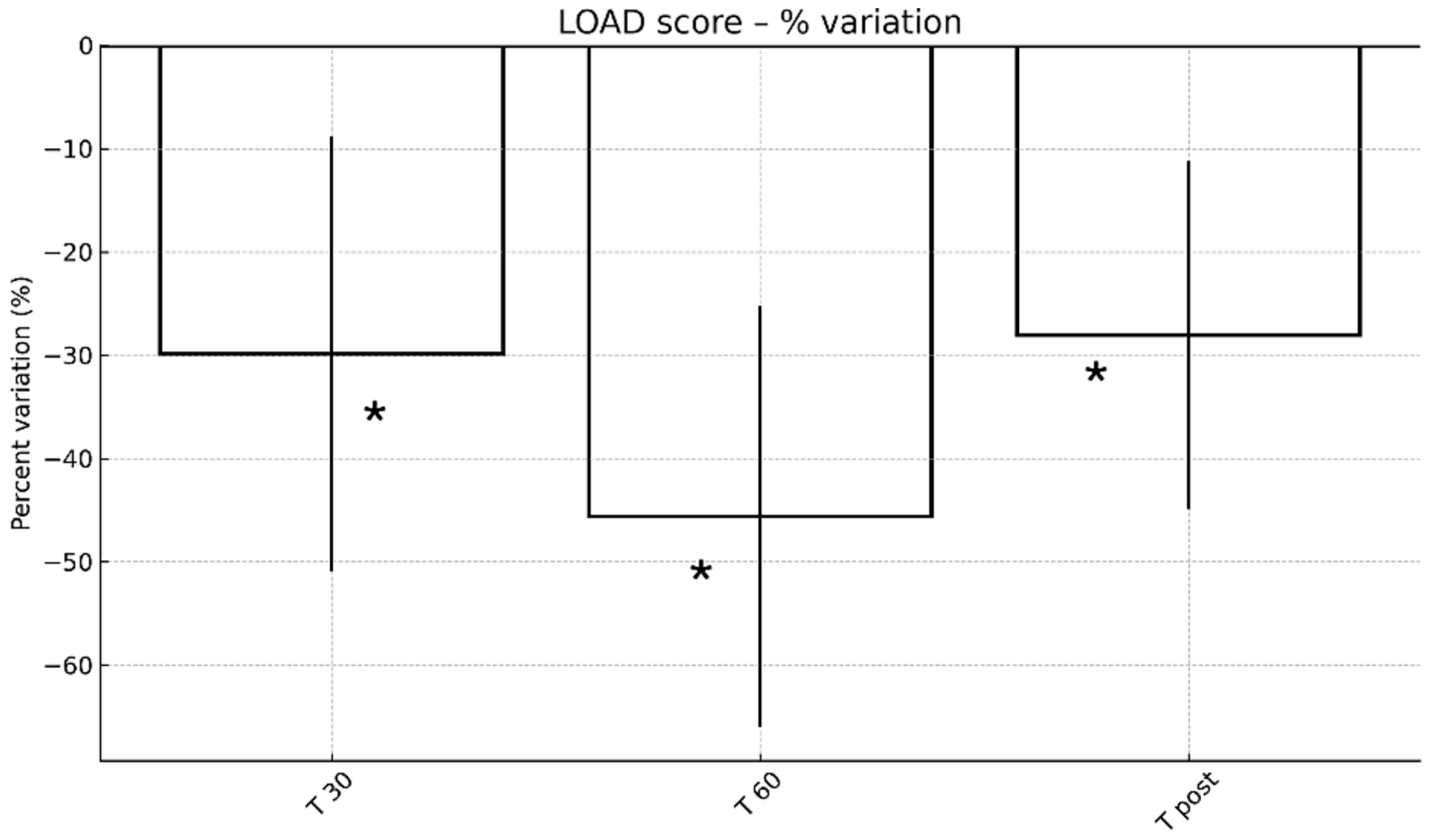
Mean percentage variation of the liverpool osteoarthritis in dogs (LOAD) score relative to baseline (Tpre) at T30, T60, and Tpost. Data are expressed as mean percentage change from baseline. *p < 0.05 compared to Tpre.

The population consisted of both male and female dogs, with an average age of 8.4 years (± 4.2). Specifically, there were 22 females and 14 males. Regarding joint involvement, the most affected site was the hip, with 46% of dogs showing bilateral hip OA. This was followed by elbow involvement in 23% of cases and knee involvement in 21%. An additional 10% of dogs presented with osteoarthritis in multiple joints in different limbs. According to the COAST scoring system for OA severity, 67% of the dogs were classified as having mild osteoarthritis, while the remaining 33% had moderate forms of the disease. Absolute values and corresponding statistical analyses of the clinical scores are reported in Table 1, while percentage variations are presented in figures to facilitate visualization of changes over time. Data related to the LOAD score showed values significantly lower at T30, T60 and Tpost compared to the value at Tpre. At T60 the score was lower than T30. Moreover, the LOAD score at Tpost was higher than the value at T60 (Table 1). The percentage variation of the LOAD score showed a reduction (*p* < 0.01) at T30 (− 29.9%), T60 (− 45.6%) and Tpost (−28.1%) compared to the Tpre time (Figure 2).

**Table 1:** Clinical scores at each timepoint in dogs included in the study.

Parameter	Tpre	T30	T60	Tpost
LOAD	32.5 (20–49)	21.5 (8–38)*	17.5 (7–36)*^,$,&^	24.0 (7–35)*
GLS	78 (62–90)	93 (81–112)*	99 (78–112)*^,$,&^	87 (71–99)
PSS	5.0 (3.7–7.5)	4.2 (0.5–6.2)*	3.2 (0.0–7.0)*	5.0 (0.5–8.0)
PIS	6.4 (3–8)	4.3 (0–6)*	3.6 (0–8)*	5.7 (0–8)
QoL	0.7 (0–2)	1.78 (0–3)*	2.3 (0–3)*	1.4 (0–3)

Data are presented as median (minimum–maximum) for Liverpool Osteoarthritis in Dogs (LOAD), gait lameness score (GLS), Pain Severity Score (PSS), Pain Interference Score (PIS), and quality of life (QoL) derived from the Canine Brief Pain Inventory (CBPI) questionnaire. Evaluations were performed at Tpre (baseline, before treatment), after 30 (T30) and 60 (T60) days of grapiprant treatment (2 mg/kg orally once daily), and 30 days after treatment discontinuation (Tpost). *p* < 0.05 compared to Tpre (*), T30 ($) and Tpost (&).

GLS score showed an improvement at T30 (*p* < 0.001) and T60 (*p* < 0.001) compared to Tpre. The GLS score at Tpost was lower that T60 (*p* < 0.001) and similar to T30 and Tpre (Table 1).

**Figure 3:**
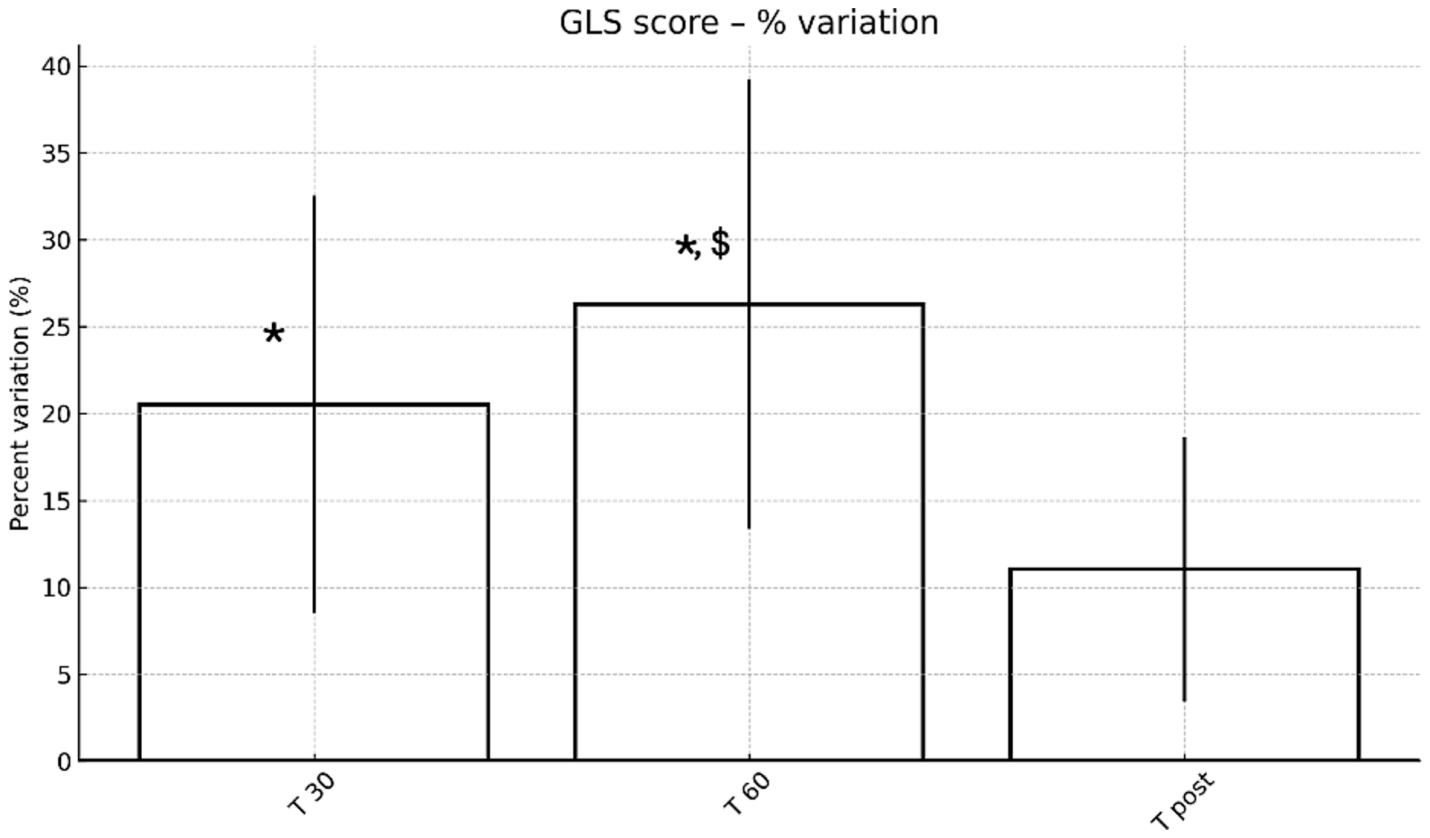
Mean percentage variation of the gait lameness score (GLS) relative to baseline (Tpre) at T30, T60, and Tpost. Data are expressed as mean percentage change from baseline. *p < 0.05 compared to Tpre; $*p* < 0.05 compared to T30.

Similarly, the percentage variation of the GLS score showed an improvement compared to Tpre at T30 (20.5%, *p* < 0.05) and T60 (26.2%, *p* < 0.001) with this last score that was higher that T30 (*p* < 0.05). The percentage variation of the GLS score at Tpost (11.1%) was reduced returning to values similar to Tpre (Figure 3).

The PSS score showed a reduction compared to Tpre at T30 (*p* = 0.004) and T60 (*p* = 0.02), returned to values similar to those observed at Tpre (Figure 2).

**Figure 4:**
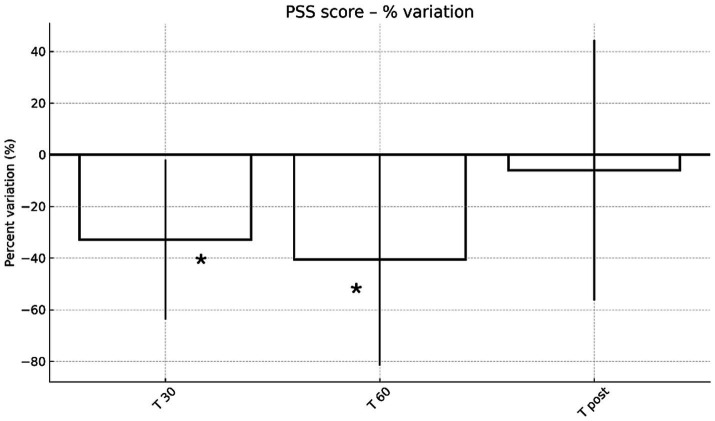
Mean percentage variation of the pain severity score (PSS) relative to baseline (Tpre) at T30, T60, and Tpost. Data are expressed as mean percentage change from baseline. *p < 0.05 compared to Tpre.

Compare to Tpre, the percentage variation of PSS showed a decrease (*p* < 0.01) at T30 (−32.9%) and T60 (− 40.6%), while at Tpost (− 5.9%) the score returned to values similar to Tpre (Figure 4).

The PIS score showed a reduction compared to Tpre at T30 (*p* = 0.017) and T60 (*p* = 0.0195). At Tpost the score was not different from Tpre (Table 1).

**Figure 5:**
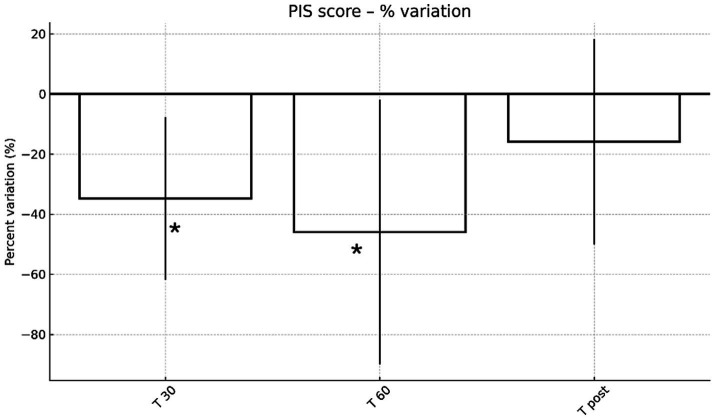
Mean percentage variation of the Pain Interference Score (PIS) relative to baseline (Tpre) at T30, T60, and Tpost. Data are expressed as mean percentage change from baseline. *p < 0.05 compared to Tpre.

The percentage variation of the PIS showed a reduction (*p* < 0.01) at T30 (−34.7%) and T60 (−46.0%) compared to Tpre (Figure 5).

The QoL score showed an improvement compared to Tpre at T30 (*p* = 0.023) and T60 (*p* = 0.0094). At Tpost the score was not different from Tpre (Table 1).

**Figure 6:**
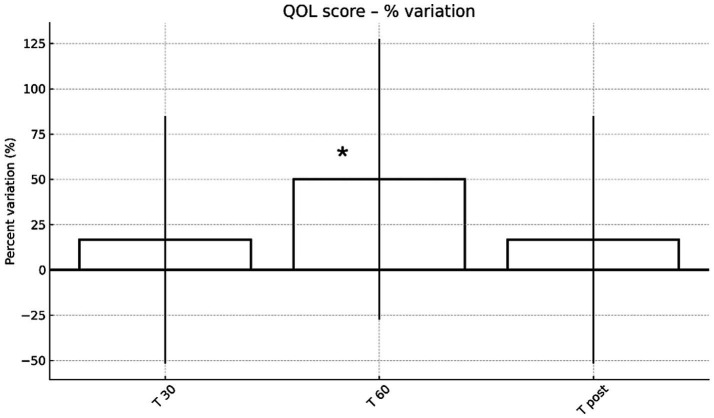
Mean percentage variation of quality of life (QoL) score relative to baseline (Tpre) at T30, T60, and Tpost. Data are expressed as mean percentage change from baseline. *p < 0.05 compared to Tpre.

The percentage variation of the QoL showed an improvement at T60 (50.0%, *p* < 0.01) compared to Tpre. At Tpost the value (16.7%) was similar to Tpre (Figure 6).

### Plasma sample analysis

**Figure 7:**
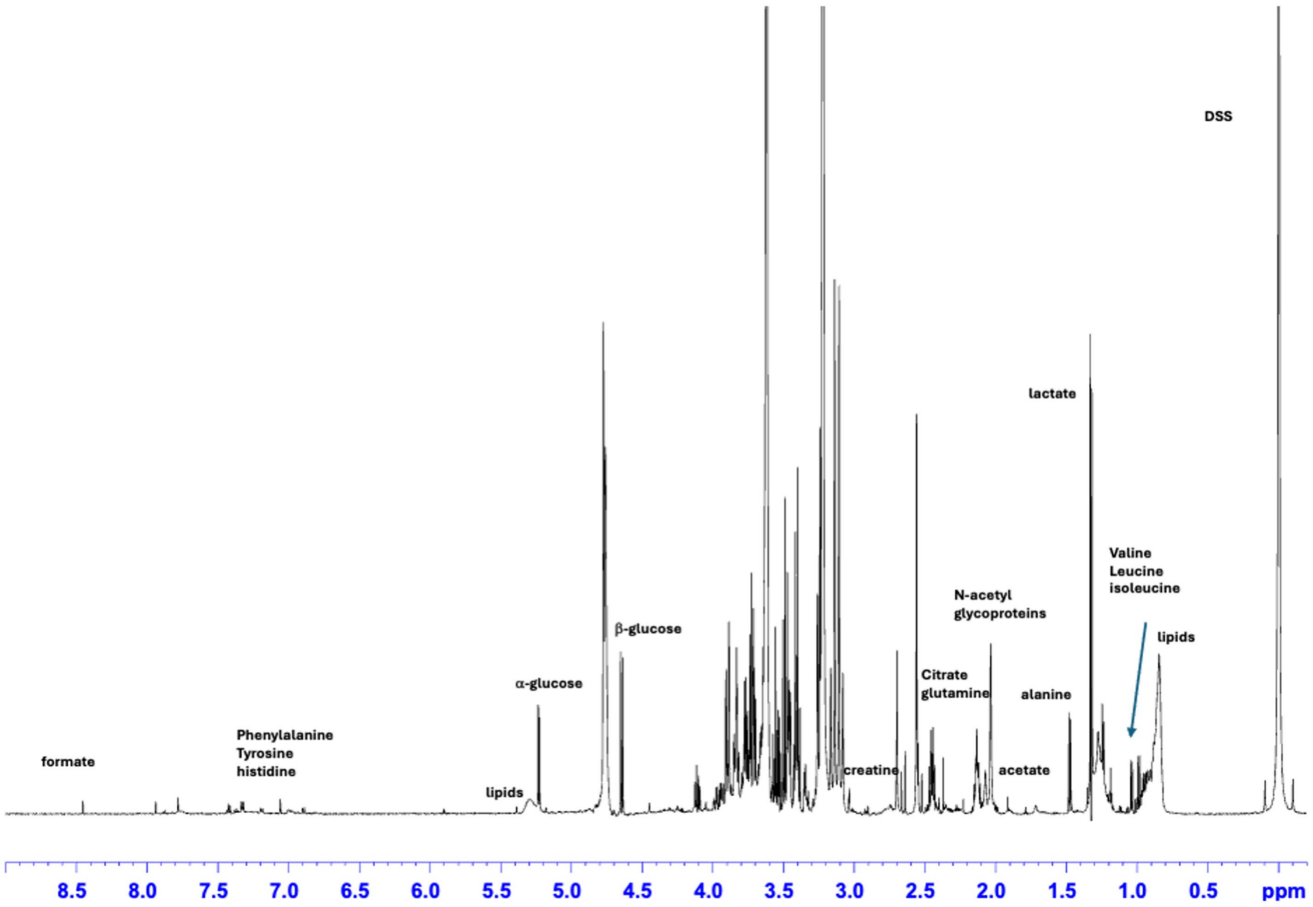
Representative ^1^H NMR spectrum of a canine plasma sample. Well-resolved and reliably assigned metabolite resonances are indicated based on spectral analysis.

**Figure 8:**
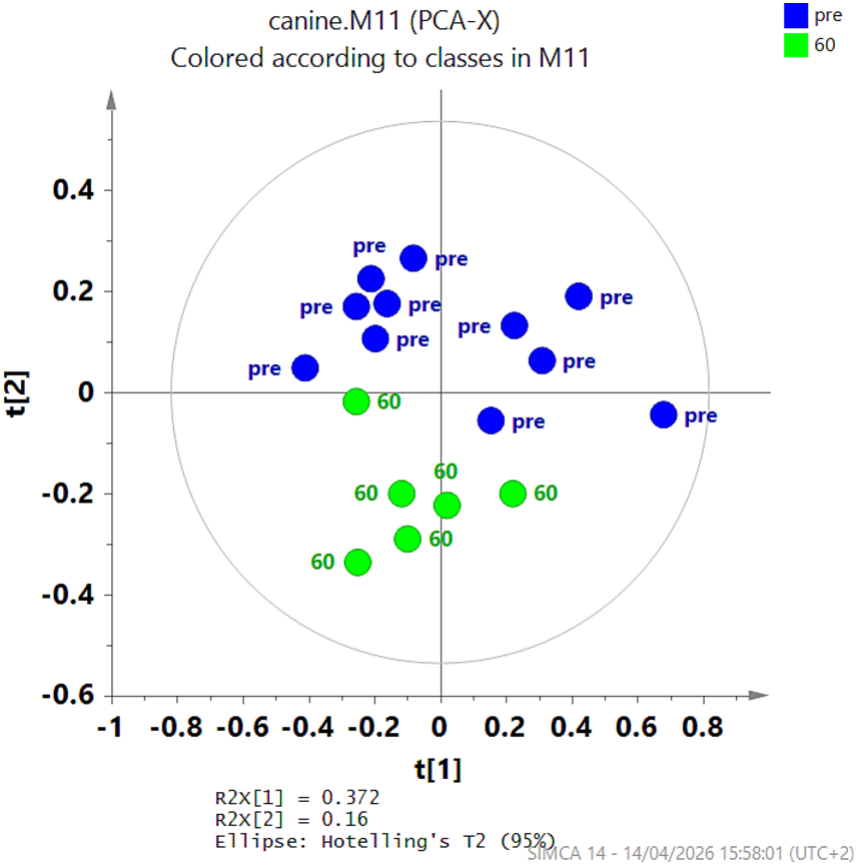
PCA t[1]/t[2] scores plot for the whole plasma samples data set. Symbols are coloured according to sampling time: Tpre and T60.

**Figure 9:**
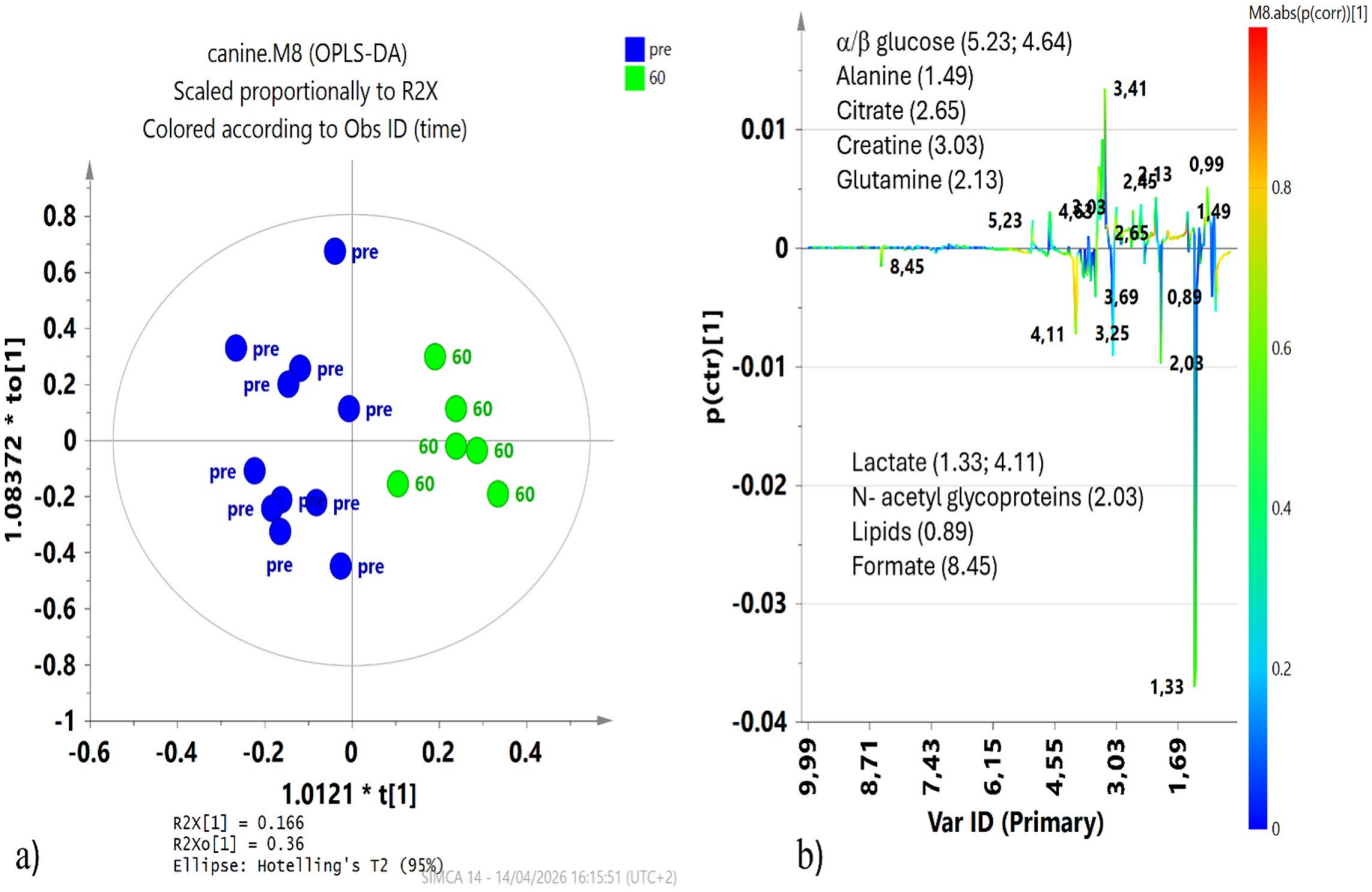
**(a)** OPLS-DA t[1]/to[1] scores plot for Tpre and T 60 class samples. **(b)** S line plot for the model colored according to the correlation-scaled coefficient [*p(corr) ≥ |0.5|]. The color bar associated with the plot indicates the correlation of the metabolites discriminating among classes.

**Figure 10:**
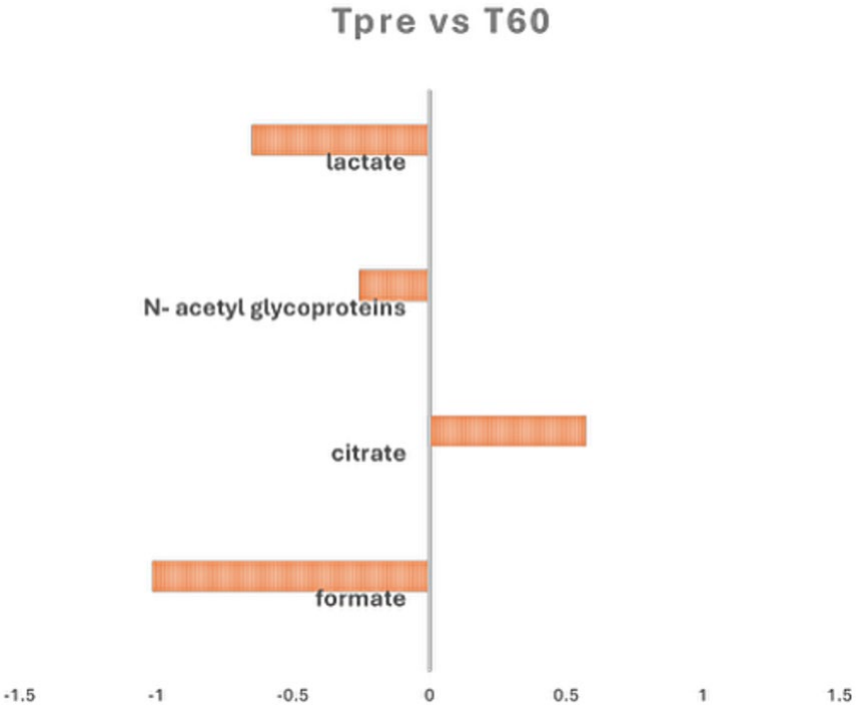
Graphic representation of discriminant metabolite comparison between Tpre and T60 plasma samples. The X-axis reports significant (*p* value < 0.05) log2 fold change (FC) values.

A representative ^1^H NMR spectrum and the assignments of clearly identifiable metabolite resonances is reported as Figure 7. Due to variability in spectral quality and suitability for multivariate analysis, only a subset of plasma samples was included in the PCA and OPLS-DA models, resulting in a reduced number of samples compared to the total study population. The ability of ^1^H-NMR-based multivariate analysis to discriminate samples from different sampling time (Tpre and T60), was employed by performing unsupervised Principal Component Analysis (PCA) and supervised Orthogonal Partial Least Square Discriminant Analysis (OPLS. DA) on the whole NMR data set. The scores plot of the preliminary unsupervised PCA analysis was built with four component, (R2X(cum) and Q2(cum) of 0.733 and 0.404, respectively) and showed, a specific separation between Tpre, and T60 plasma samples along the second principal component (Figure 8). Then a supervised OPLS-DA (one predictive and three orthogonal components, 1 + 3 + 0) was applied to refine the observed separation and to identify the discriminating molecular components. The resulting OPLS-DA model, was described by good predictive (Q2 = 0.621) and descriptive (R2X and R2Y of 0.526 and 0.835, respectively) parameters. Since both values exceeded 0.5, the model can be considered to fit the data well and with good predictive ability. The permutation test (200 iterations) yielded intercepts of R2 = 0.594 and Q2 = −0.635, indicating no evidence of overfitting and a clear separation between classes (Supplementary Figure S1). Moreover, the CV-ANOVA (ANOVA assessment of the cross-validatory (CV) predictive residuals) yielded a *p* value = 0.014, confirming the significance of model. The resulting scores plot showed a clear discrimination between Tpre and T60 plasma samples (Figure 9a). The corresponding S line plot of the loading vectors for the first components, coloured according to the pcorr values, showed the molecular components responsible for the observed separation among the samples (Figure 9b). Samples of Tpre class, representative of dogs before the treatment, were characterized by higher relative content of lactate (bins at 1.33, 4.11 ppm), N- acetylglycoprotein (bin at 2.03), lipids (bin at 0.89) and formate (bin at 8.45 ppm). On the contrary, after the treatment, T60, samples exhibited higher content of a/b glucose (5.23 and 4.64 ppm, respectively), creatine (3.03 ppm), citrate (2.65 ppm), glutamine (bin at 2.13 ppm) and alanine (1.49 ppm). Potential significant discriminating metabolites were further selected: only assigned metabolites showing a major contribution to the OPLS-DA models, with high statistical reliability |p(corr)| ≥ 0.5 and strong discrimination power (VI*P* ≥ 1), were considered for relative quantification. The change in the selected assigned discriminating metabolites was then calculated as the ratio of the standardized median intensity the NMR signal corresponding to selected buckets (fold change, FC). FCs were calculated from the buckets’ integral values of the unbiased NMR signals for lactate, N-acetyl glycoproteins, citrate, and formate. The statistical significance of the differences for each variable of the two classes in the pairwise comparison was evaluated using the student t-test (*p* value < 0.05). (Table 2; Figure 10). Significant log2(FC) values are reported in the graph.

**Table 2:** Fold change (FC) ratios of the discriminant metabolites between the two group means (T60/Tpre).

Metabolites	Chemical shift (ppm)	FC (T60/Tpre)	LOG2 FC	*p*-value (t-test)
Lactate	1.33	0.64	−0.65	0.04
N-acetyl glycoproteins	2.03	0.84	−0.26	0.04
Citrate	2.65	1.49	0.57	0.026
Formate	8.45	0.49	−1.01	0.015

Significance of the values from univariate t- test is reported.

Metabolites contributing to the separation observed in the OPLS-DA model were identified as discriminant; however, not all of these showed statistically significant differences in univariate analysis.

## Discussion

This prospective within-subject, off–on–off clinical study assessed the efficacy of grapiprant, an EP4 prostaglandin receptor antagonist, in improving clinical signs of osteoarthritis in dogs, alongside its impact on systemic metabolomic profile. The multimodal assessment, which included owner-reported pain and mobility scores (LOAD, CBPI), gait analysis (GLS), and advanced plasma metabolomics, allowed for a comprehensive evaluation of treatment response.

### Clinical impact of grapiprant on OA signs

Significant clinical improvements were observed following grapiprant administration. The LOAD score, a validated tool for owner-reported mobility impairment (21), showed a statistically significant decrease during treatment (T30 and T60) compared to the pre-treatment phase (Tpre). Notably, although LOAD scores increased slightly post-treatment (Tpost), they remained significantly lower than baseline, suggesting a residual therapeutic effect even after discontinuation. The reduction in LOAD largely exceeded the 20% threshold considered clinically meaningful for this score (22). Similarly, the GLS (Gait Lameness Score), an objective measure of limb loading during ambulation, improved progressively during treatment. However, the reduction in GLS post-treatment mirrored the LOAD trends, indicating a return toward baseline once the pharmacologic effect ceased.

Together, these results confirm the beneficial analgesic efficacy of grapiprant over a two-month treatment period in dogs with mild to moderate OA. The partial regression of benefits post-treatment underscores the need for continuous administration in chronic OA management, as expected from a drug targeting symptomatic rather than structural disease pathways (5).

The metabolomic analysis performed through 1H NMR spectroscopy revealed significant alterations in the plasma metabolic profile of canine subjects between the pre-treatment (Tpre) and after 60 days of treatment (T60). The multivariate approach enabled a comprehensive understanding of the treatment effects, showing a separation between the two groups using PCA and OPLS-DA. Notably, the OPLS-DA model demonstrated strong performance parameters (R^2^Y = 0.835; Q^2^ = 0.621), supporting the presence of distinct metabolic patterns associated with the treatment.

From a biological perspective, Tpre samples showed relatively higher concentrations of lactate, formate, lipids, and N-acetyl glycoproteins. These metabolites are known to be associated with inflammatory states, oxidative stress, and disruptions in energy metabolism (30). Elevated lactate levels, for instance, may indicate increased anaerobic glycolysis, typically found in hypoxic or inflammatory conditions (17). Similarly, N-acetyl glycoproteins are widely recognized as biomarkers of systemic inflammation, while increased formate concentrations may suggest mitochondrial dysfunction or stress (11, 31).

In contrast, the T60 samples exhibited increased levels of glucose (*α* and *β*), creatine, citrate, glutamine, and alanine, suggesting a potential shift toward improved mitochondrial metabolic function and an improvement in overall physiological condition (32, 33). Citrate and alanine are directly involved in the Krebs cycle and gluconeogenesis, respectively, which are essential for efficient energy metabolism (11, 31, 34). Although not statistically significant (*p* = 0.24), creatine is an important marker for muscle function and short-term energy storage, indicating improved bioenergetic status in the treated dogs (18). While increased creatine levels may be associated with renal function, no clinical or laboratory evidence of renal impairment was observed in the study population.

Univariate analyses identified lactate, formate, and N-acetyl glycoproteins (decreased post-treatment), and citrate (increased post-treatment) as significantly altered metabolites (*p* < 0.05). The reduction of inflammatory markers and the increase of metabolites related to efficient energy metabolism support the hypothesis of a beneficial effect of the treatment (30, 35). In this study, the intervention consisted of the administration of grapiprant that selectively antagonizes the EP4 receptor, a key mediator of pain and inflammation in osteoarthritis (36).

These findings are consistent with previous observations in similar contexts, where pharmacological interventions led to favorable modifications in the metabolic profiles of treated animals (11, 14, 37). Moreover, the analytical procedures employed, including normalization and Pareto scaling, have been shown to enhance biological interpretability in metabolomic studies (29). The statistical robustness of the models further supports the reliability of the findings (16).

The metabolomic analysis enabled the identification of key markers associated with the metabolic response to treatment with grapiprant in dogs. The findings suggest a potentially positive effect of the intervention, associated with reduced inflammation and improved energy metabolism.

The study from Piccionello et al. (37) investigated the 1H-NMR metabolomic profile of canine synovial fluid across progressive stages of spontaneous osteoarthritis. Distinct metabolic shifts were identified, with early OA characterized by higher mannose, betaine, and 2-hydroxyisobutyrate, and advanced OA showing increased isoleucine and lactate. These findings indicate progressive alterations in energy, amino acid, and inflammatory metabolism.

This study is the first to integrate clinical scores, objective gait analysis, and untargeted pharmacometabolomics to evaluate grapiprant in a naturally occurring OA canine model. The observed metabolic shifts may support the hypothesis that grapiprant exerts symptomatic relief and could be associated with modulation of systemic metabolic pathways associated with OA pathophysiology.

From a translational perspective, the study demonstrates the feasibility and value of metabolomic biomarkers in monitoring treatment response and potentially identifying metabolic endotypes of OA. In the future, this approach could guide individualized therapy and help distinguish responders from non-responders to specific anti-inflammatory agents.

Limitations include the finite duration of the off–on–off design, which does not address long-term metabolic trajectories. Metabolomic sampling was limited to selected timepoints due to ethical considerations and the lack of established knowledge on metabolite kinetics, reflecting the exploratory nature of the study. In addition, metabolomic analysis was performed on a subset of samples meeting quality criteria, which may limit the generalizability of the findings; however, the consistency of the multivariate models suggests that the observed metabolic patterns are robust (Supplementary Figure S1). A subgroup analysis based on osteoarthritis severity was not performed due to the limited sample size within each subgroup, which may have reduced the statistical robustness of such comparisons. Additional limitations include the absence of a control group, the relatively small sample size, and the potential risk of overfitting in multivariate analyses, despite the validation procedures applied. Although several factors such as body weight, body condition score, age, sex, and lifestyle variables may influence clinical outcomes in osteoarthritis, their impact is difficult to quantify in a clinical setting, particularly in the absence of well-defined phenotypic stratification in veterinary patients. These aspects should therefore be considered as limitations of the present study.

Future studies should employ larger cohorts, longer treatment periods, and multi-omics integration to refine biomarkers of response and guide patient stratification.

In conclusion, grapiprant improved pain and mobility and induced systemic metabolic changes consistent with lowered inflammation and improved bioenergetic status. This is, to our knowledge, the first study integrating clinical outcomes with untargeted metabolomics in canine OA treated with grapiprant, underscoring the translational value of metabolomics for monitoring therapeutic response.

## Data Availability

The raw data supporting the conclusions of this article will be made available by the authors, without undue reservation.
