# Supplementary material for: Evaluation of clinical effects and plasma metabolomic profiles after the administration of grapiprant in dogs affected by osteoarthritis: a prospective, off–on–off, clinical study
Source: Front Vet Sci. 2026 Jun 15;13:1811478. doi: 10.3389/fvets.2026.1811478 (PMC13312814; doi:10.3389/fvets.2026.1811478)
Supplement: Supplementary file 1 [file Data_Sheet_1.pdf]

Supplementary material

Evaluation of clinical effects and plasma metabolomic profiles after the administration of grapiprant in dogs affected by osteoarthritis: a prospective, off-on–off, clinical study

Piemontese C[1]., Chiara Roberta Girelli[2], Marzia Stabile[1], Luca Lacitignola[1], Agata Fraccascia[1], Antonio Crovace[1], Francesco Paolo Fanizzi[2], Francesco Staffieri[1]

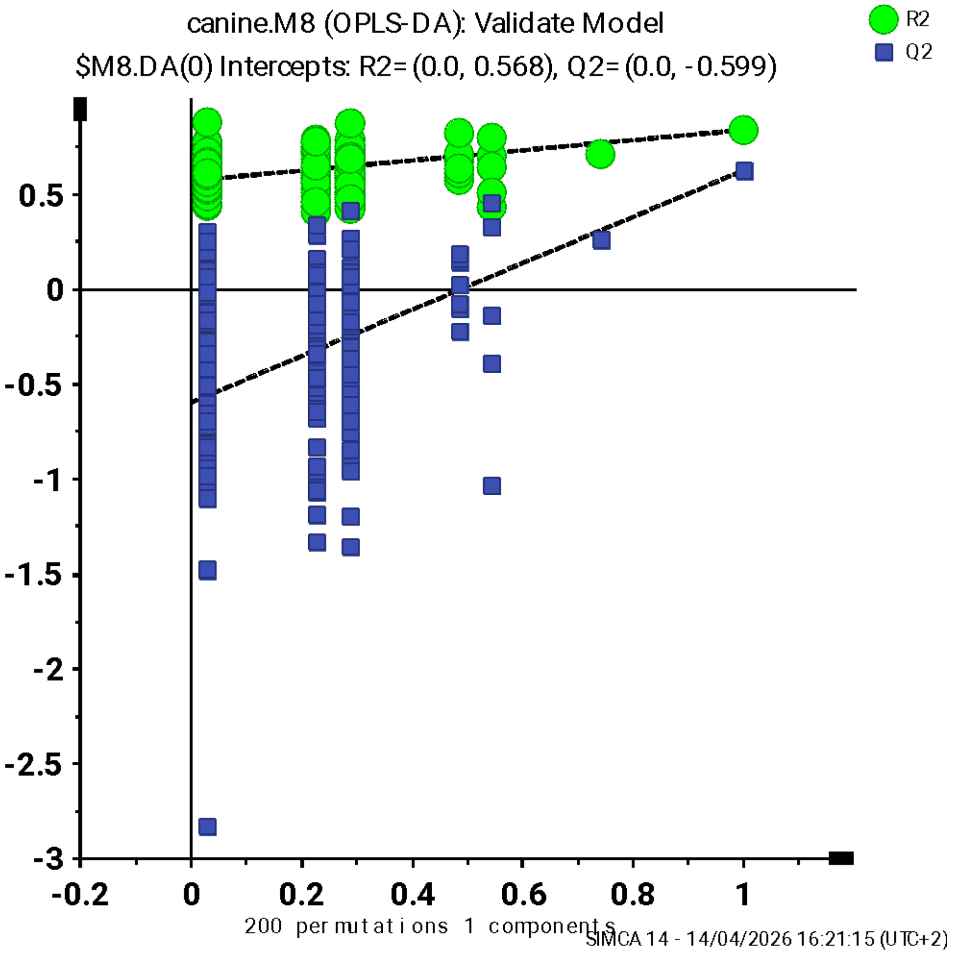

Fig. S1 – Validation plot of 200 permutation tests for OPLS-DA model built for Tpre and T60 serum samples. Q2 -intercept of −-0.599 is less than 0.05 indicating a valid model.

Table S1 List of discriminating chemical descriptors (variables) with corresponding correlation coefficient (pcorr), and variable importance on the projection (VIP) for Tpre and T60 serum samples. Metabolites with high statistical reliability |p(corr)| ≥ 0.5 and strong discrimination power (VIP ≥ 1), were considered for relative quantification.

Assigned Metabolite	Var ID (Primary)	M8.p(corr) [1]	M8.VIP [1+1+0]
**Formate**	**8.45**	-0.48	1.07062
**α-glucose**	**5.23**	0.31	1.72618
**β-glucose**	**4.65**	0.30	1.55166
**creatine**	**3.03**	0.30	1.4333
**citrate**	**2.65**	0.58	1.7027
**glutamine**	**2.13**	0.39	2.04552
**N- acetyl glycoproteins**	**2.03**	-0.57	3.08719
**alanine**	**1.49**	0.36	1.28307
**lactate**	**1.33**	-0.56	6.44528
**lipids**	**0.89**	-0.08	3.8275

Table S2. Individual characteristics of the included dogs, including signalment, body weight, body condition score (BCS), and osteoarthritis classification.

Dog ID	Age (years)	Sex (M/F)	Breed	Body weight (kg. Tpre)	Body weight (kg. Tpost)	BCS (Tpre)	BCS (Tpost)	OA location	COAST stage
1	9.4	F/N	German S.	34.8	33.1	5	5	Hip	Mild
2	4.3	F/I	Mixed Breed	18.6	17.2	6	6	Knee	Mild
3	12.7	M/I	German S.	37.2	38.5	6	6	Hip	Mild
4	7.1	F/N	Spinone I	31.5	27.3	7	7	Knee	Mild
5	5.9	M/N	Jack Russell	7.4	7.8	5	5	Mult. Joints	Moderate
6	13.3	M/N	German S.	32.9	33.6	5	5	Hip	Mild
7	3.9	F/N	Mixed Breed	12.8	13.7	5	5	Elbow	Mild
8	10.6	F/N	Golden R.	29.6	31.5	6	6	Hip	Mild
9	6.8	M/I	Amstaff	27.3	28.2	8	8	Hip	Moderate
10	11.5	F/N	Mixed Breed	21.4	20.7	8	8	Knee	Moderate
11	4.7	F/I	Golden R.	31.2	33.2	7	7	Elbow	Mild
12	8	F/N	Amstaff	24.9	21.4	6	6	Hip	Mild
13	12.1	M/N	Mixed Breed	9.7	10.2	5	5	Elbow	Mild
14	5.1	F/N	Cocker	13.8	6.8	4	4	Hip	Mild
15	9.7	M/I	Golden R.	28.4	26.1	5	5	Elbow	Moderate
16	13.6	F/N	Mixed Breed	16.5	17.3	7	7	Hip	Mild
17	6.2	F/N	Golden R.	33.1	33.8	6	6	Elbow	Mild
18	10.3	F/N	Rottweiler	43.6	41.4	5	5	Hip	Mild
19	3.7	M/I	Golden R.	30.5	31.2	5	5	Mult. Joints	Mild
20	11.2	F/I	Jack Russell	6.8	7.2	7	7	Hip	Moderate
21	7.7	M/I	Mixed Breed	24.2	22.9	6	6	Hip	Mild
22	4.5	M/I	Amstaff	29.1	27.8	7	7	Elbow	Moderate
23	12.4	F/N	Golden R.	27.8	28.3	5	5	Elbow	Mild

24	8.8	M/N	Collie B	18.9	18.5	6	6	Hip	Moderate
25	5.3	F/I	Mixed Breed	14.3	15.9	5	5	Knee	Moderate
26	14	M/N	Golden R.	32.4	33.2	6	6	Elbow	Mild
27	6.5	F/N	Cocker	15.1	15.3	7	7	Hip	Moderate
28	9.1	F/N	Amstaff	25.7	26.6	8	8	Hip	Mild
29	4.1	M/N	Mixed Breed	7.9	8.9	6	6	Mult. Joints	Moderate
30	10.9	F/I	Collie B	20.6	22.1	7	7	Knee	Mild
31	7.4	F/N	Rottweiler	45.0	44.8	7	7	Elbow	Moderate
32	13	M/I	Mixed Breed	19.8	18.2	8	8	Hip	Mild
33	5.6	F/N	Cocker	12.6	14.3	8	8	Knee	Moderate
34	11.8	F/N	Golden R.	29.3	27.9	6	6	Mult. Joints	Mild
35	4.9	M/N	Collie B	17.2	18.2	5	5	Knee	Mild
36	3.8	F/N	Mixed Breed	11.4	12.2	7	7	Hip	Mild
